# Detection of response shift in health-related quality of life studies: a systematic review

**DOI:** 10.1186/s12955-022-01926-w

**Published:** 2022-02-05

**Authors:** Estelina Ortega-Gómez, Purificación Vicente-Galindo, Helena Martín-Rodero, Purificación Galindo-Villardón

**Affiliations:** 1 Department of Statistics, University of Panama, Simon Bolivar Ave., Octavio Mendez Pereira Campus, Panama City, Republic of Panama; 2 Department of Statistics, University of Salamanca, 37008 Salamanca, Spain; 3 Department of Statistics, IBSAL, University of Salamanca, Salamanca, Spain; 4 Department of Library and Information, University of Salamanca, Alfonso X El Sabio St. 37007, Miguel de Unamuno Campus, Salamanca, Spain; 5 Centro de Investigación Institucional (CII), Universidad Bernardo O’Higgins, Avenida Viel 1497, Santiago, Chile; 6 Universidad Estatal de Milagro, Guayas, Ecuador

**Keywords:** Systematic review, Response shift, Multivariate analysis, Statistical methods

## Abstract

**Background:**

Response Shift (RS) refers to the idea that an individual may undergo changes in its health-related quality of life (HRQOL). If internal standard, values, or reconceptualization of HRQOL change over time, then answer to the same items by the same individuals may not be comparable over time. Traditional measures to evaluate RS is prone to bias and strong methodologies to study the existence of this phenomenon is required. The objective is to systematically identify, analyze, and synthesize the existing and recent evidence of statistical methods used for RS detection in HRQOL studies.

**Methods:**

The analysis of selected studies between January 2010 and July 2020 was performed through a systematic review in MEDLINE/PubMed, Scopus, Web of Science, PsycINFO and Google Scholar databases. The search strategy used the terms “Health-Related Quality of Life” and “Response Shift” using the filters “Humans”, “Journal Article”, “English” and “2010/01/01–2020/07/31”. The search was made in August 2020.

**Results:**

After considering the inclusion and exclusion criteria, from the total selected articles (675), 107 (15.9%) of the publications were included in the analysis. From these, 79 (71.0%) detected the existence of RS and 86 (80.4%) only used one detection method. The most used methods were Then Test (*n* = 41) and Oort’s Structural Equation Models (SEM) (*n* = 35). Other method used were Multiple Lineal Regression (*n* = 7), Mixed-Effect Regression (*n* = 6), Latent Trajectory Analysis (*n* = 6), Item Response Theory (*n* = 6), Logistics Regression (*n* = 5), Regression and Classification Trees (*n* = 4) and Relative Importance Method (*n* = 4). Most of these detected recalibration, including Then Test (*n* = 27), followed by Oort’s SEM that detected the higher combination of RS types: recalibration (*n* = 24), reprioritization (*n* = 13) and reconceptualization (*n* = 7).

**Conclusions:**

There is a continuous interest of studying RS detection. Oort’s SEM becomes the most versatile method in its capability for detecting RS in all different types. Despite results from previous systematic reviews, same methods have been used during the last years. We observed the need to explore other alternative methods allowing same detection capacity with robust and highly precise methodology. The investigation on RS detection and types requires more study, therefore new opportunity grows to continue attending this phenomenon through a multidisciplinary perspective.

**Supplementary Information:**

The online version contains supplementary material available at 10.1186/s12955-022-01926-w.

## Background

Health-related quality of life (HRQOL) is the affectation of a patient’s physical and mental health, and their correlates, including health risk and conditions, independence level, social support, and socioeconomic status over time [1, 2].

This concept is established as a facet of an individual’s state of life while measuring its well-being as a medical patient. HRQOL is analyzed as a functional health state and identifies effective strategies to improve patient´s conditions as a result of medical interventions [3, 4].

After a medical treatment, patients may perceive and demonstrate different conditions over time, from the procedure’s initial stages up to many years after treatment has finished. The changes in the measurement of individual’s perception or internal standard is known as Response Shift (RS) [5, 6]. From a clinical perspective, RS is generated as the change in the meaning of a subject self-administrated assessment [7] as a valid and sensitive mechanism to evaluate the change in different moments in time [8].

Spranger and Schwartz [9] theoretical model explains how RS may affect HRQOL as a result of health state changes. As a baseline model, it presents five components: (1) a catalysts, corresponding to an individual health states or its changes as a result or not of a treatment; (2) antecedents, referring to individual’s characteristics influencing catalysts or appraisals mechanisms; (3) mechanism, explained by behavioral, cognitive, or affective processes accommodating changes in catalysts; (4) response shift, representing changes in the meaning of an individual self-evaluation of QOL resulting from changes in internal standards, values, or conceptualization; and (5) perceived QOL. Rapkin and Schwartz [10] propose that QOL appraisal processes must consider how individuals perceive their health status and respond questionnaires about their QOL. The model follows these processes: (1) induction of a frame of reference; (2) sampling based on the frame of reference; (3) judge against standards of comparison; and (4) combine algorithms to formulate a response. The proposal allows dynamic feedback to explain how QOL scores can be stabilized accounting inter-individual and temporary differences despite changes in health status [10].

An individual’s self-assessment may demonstrate changes in three contexts: in the internal standards of the measurement scale (recalibration) indicating that the patient has a new scale for measuring its own state of HRQOL; in the scale of values (reprioritization) representing a change in the priority of elements that influence the context of life; and in the definition of the objective construct (reconceptualization) when a patient raises a redefinition of its own concept [9].

The change processes of a patient must be appropriately measured when the effects of a disease or medical treatments is evaluated [11, 12]. The interpretation of HRQOL data represents a challenge because patients self-report their health conditions at a specific time, which can also be influenced by psychological phenomena [13]. This suggests that HRQOL measurements must consider that the individual reports on its status, at least in two or more moments to detect significant changes over time.

Two main approaches are proposed for the detection of change: methods based on specific study designs and secondary data analysis that includes statistical methods developed to test hypotheses that do not require specific designs [14].

The most commonly used methodology is the retrospective Then Test design [15] that allows evaluating the change of patient´s internal standard by comparing the scores with two other moments: pre-test and post-test [5, 7]. However, this method is sensitive to bias and difficult to be used in longitudinal secondary data analysis [15].

Structural Equation Models (SEM) of Schmidt [16] and Oort [17], Item Response Theory (IRT) of Anota et al. [18], and Guilleaux et al. [19], Relative Importance Method of Lix et al. [20], Latent Trajectory Modelling of Ahmed et al. [21], and Classification and Regression Trees of Li & Schwartz [22] are among the most widely used and proven methods for RS detection in both primary and secondary data.

This bibliographic review has permitted to identify the methods traditionally applied in specific clinical studies related to HRQOL or to the analysis of previously elaborated databases, mainly by medical, academic or research institutions. Although other recent publications have carried out exhaustive systematic reviews to address this issue [15, 23], it is necessary to continue exploring if other alternative statistical methods are used as emerging mechanisms in the RS detection. Consequently, the purpose of this investigation is to systematically identify, analyze, and synthesize the existing evidence of new statistical methods used for RS detection in HRQOL studies.

## Methods

The systematic review is a structured methodology allowing the identification and integration of different specific studies based on inclusion and exclusion criteria and facilitates the eligibility of relevant or interesting publications [24]. Some critical and determining elements to capture the largest number of eligible publications are the number and orientation of search repositories, and the established inclusion and exclusion criteria.

The PRISMA methodology [25] was applied to develop this systematic review. Through pre-specified eligibility criteria, it allows reducing bias in the identification, selection, synthesis, and summary of results from previously published studies [26]. The precision and reliability of this tool provides important benefits in health-related research [27].

The acceptance of this methodology has been used in recent studies on RS applied to different clinical areas: oncology [23, 28], orthopedic rehabilitation [29], pre-injury [30], as well as patient-reported outcomes (PRO) [31].

### Data sources

The selected studies were carried out through an organized review of MEDLINE/PubMed, Scopus, Web of Science Core Collection (SSCI) PsycINFO and Google Scholar databases. The terms considered in MEDLINE/PubMed search are “Quality of Life”, “Health-Related Quality of Life”, “Response Shift” in descriptors or keywords in the title and/or abstract. The search equation was “Quality of Life”[Mesh] OR “Quality of Life”[Title/Abstract] OR “Life Quality”[Title/Abstract] OR “Health-Related Quality of Life”[Title/Abstract] OR “Health Related Quality of Life”[Title/Abstract] OR “QoL” [Title/Abstract] OR “HRQoL”[Title/Abstract]) AND "Response Shift"[Title/Abstract].

The following filters were used: “Humans”, “Journal Articles”, “English”, “2010/01/01–2020/07/31". This search strategy was adopted for each of the databases consulted. The search was made in August 2020 and completed with an analysis of the selected literature between January 2010 and July 2020.

### Articles selection

Articles that qualified for the eligibility review were those in English language that met the following criteria: adapt to the objectives of the search to identify and synthesis of statistical methods used in RS detection in HRQOL studies; be published in peer-reviewed journals and be able to retrieve the full text of the work; and the term “Response Shift” was included in the title, abstract and/or keyword; those studies in a language other than English that did not include the use of statistical methods for the RS detection, as well as studies whose main objective was not the detection of SR and its classification. Conferences, editorial notes, systematic reviews, and conceptual evaluations were excluded from the study.

**Fig. 1:**
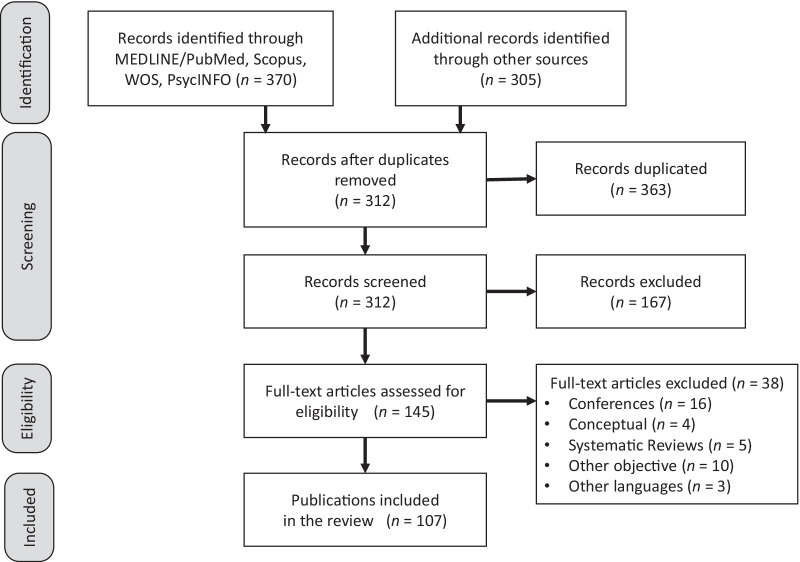
PRISMA flow diagram of the search strategy for publications included in the review

Article selection was independently made by two authors (EOJ and PVG) who initially review titles, then abstracts, and read the full texts. For article inclusion, a concordance assessment between authors was established to be greater than 80% (Kappa index). In case of discrepancies, the process was repeated until they were resolved by consensus among all the authors. Figure 1 illustrates the literature search and publication selected through the PRISMA flow diagram.

## Results

### Descriptive characteristics of the study

The search strategy identified a total of 675 articles in the databases of MEDLINE/PubMed (141), Scopus (46), Web of Science Core Collection (114), PsycINFO (69) y Google Scholar (305). After eliminating 363 duplicated records, 167 publications without the term “response shift” in the fields title, abstract and/or keyword, and other 38 articles excluded for not meeting the inclusion criteria, a total *n* = 107 (15.9%) were included in the analysis (See Additional file 1).

**Fig. 2:**
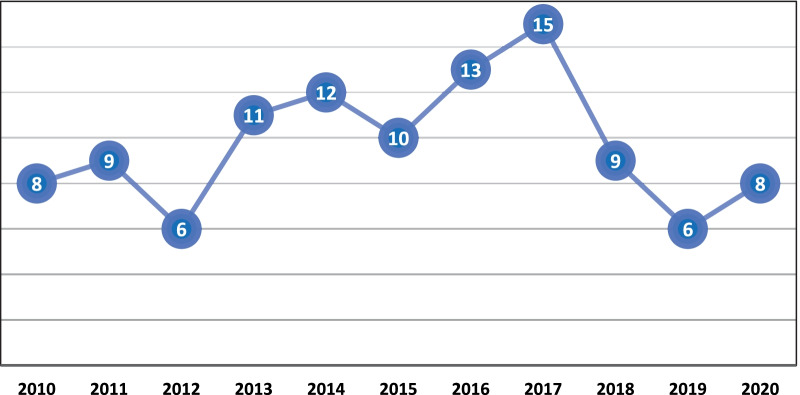
Number of publications by year

Based in the year of publication, 15 studies were recorded in 2017, followed by 2016 (13) and 2014 (12). An active interest in this research topic was continued during the rest of the years studied (see Fig. 2). A total of 56 journals were identified in this systematic review, where 31.8% of the articles were published in Quality of Life Research (34), followed by Health and Quality of Life Outcomes (10), Journal of Clinical Epidemiology (4) and European Journal of Cancer Care (4).

The analysis of the included articles determined that 69.2% of the studies were based on primary data, 29.0% on secondary data, and 1.8% did not specify. Different questionnaires were used to capture patient´s information. Some studies concentrated in the use of one instrument, while other indicated diverse mechanisms to collect data (see Table 1). The medical orientations of the publications were mainly in Oncology (27), Neurology (11), Psychology/Psychiatrics (10), Orthopedics (9), Oral health (7) and Cardiology (6).

**Table 1:** Instruments mostly used in analyzed studies

Instrument used	Code	Number
European Organisation for Research and Treatment of Cancer Quality-of-life-Questionnaire Core 30	EORTC QLQ-C30	20
36-Item Short Form Survey	SF-36	22
12-Item Short Form Survey	SF-12	7
EuroQol – 5 Dimensions	EQ-5D	10
Oral Health Impact Profile	OHIP	6

### About the methods for Response Shift detection

**Fig. 3:**
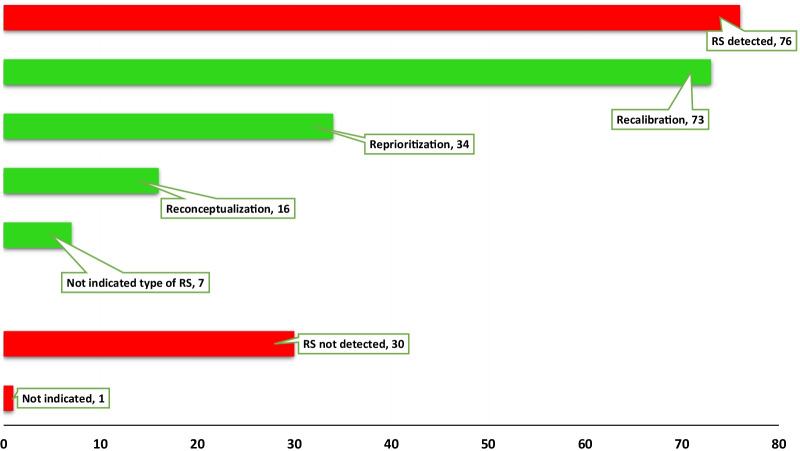
Number of articles (*n* = 107), Response Shift detection and type

Of the 107 articles analyzed, 76 (71.0%) detected the existence of RS, 30 (28.0%) did not identify it, while one article did not indicate it (see Fig. 3). The review described the methods for RS detection, types of RS, and if studies detected or not its existence. A group of 86 (80.4%) articles used one method for RS detection, 19 (17.8%) articles used two or more methods, and two articles did not specify the method used.

Table 2 describes the frequency of methods used in these publications. 41 (31.8%) articles used the Then-Test method, 35 (27.1%) adopted the Oort’s SEM and 2 (1.6%) articles indicated the use of Schmidt’s SEM. Other models were Multiple Linear Regression (7), Mixed-Effects Regression (6), Latent Trajectory Analysis (6), Item Response Theory (6), Logistic Regression (5), Classification and Regression Tree (4) and Relative Importance Method (4). Other were used but in a smaller number of studies.

**Table 2:** Frequency of methods used for Response Shift detection

Method	Total (*n* = 129)	Response shift detection		
		Yes	No	Not indicated
Then-test	41	32	9	0
Structural Equation Models (Oort)	35	26	9	0
Structural Equation Models (Schmidt)	2	2	0	0
Multiple Linear Regression	7	4	2	1
Relative Importance Method	4	3	1	0
Mixed-Effects Regression	6	5	1	0
Classification and Regression Trees	4	4	0	0
Random Forest Regression	2	2	0	0
Logistics Regression Model	5	1	4	0
Item Response Theory	6	3	3	0
Latent Trajectory Analysis	6	2	4	0
Other methods	11	7	3	1

According to the detection methods used in all articles, 91 (70.5%) detected RS, while 36 (27.9%) indicated its absence. In the review, 2 (1.6%) articles did not identify the presence or absence of this phenomenon. Of the studies that detected RS through different methods, 73 indicated the presence of recalibration, 34 indicated the existence of reprioritization, 16 highlighted reconceptualization, and 7 articles detected the existence of RS, but did not specify the type (see Table 3). From these, 41 articles detected one type of RS, 20 articles registered two types of RS, only 7 studies identified the three types simultaneously. Seven articles did not indicate it.

**Table 3:** Number of articles according to the detection method and type of Response Shift

Method	Type of Response Shift			
	Recalibration	Reprioritization	Reconceptualization	Not indicated
Then-Test	27	6	3	3
Structural Equation Models (Oort)	24	13	7	1
Structural Equation Models (Schmidt)	2	0	0	0
Multiple Linear Regression	2	0	1	1
Relative Importance Method	0	3	0	0
Mixed-Effects Regression	3	2	0	1
Classification and Regression Trees	4	2	1	0
Random Forest Regression	1	1	0	0
Logistics Regression Model	1	1	1	0
Item Response Theory	3	3	1	0
Latent Trajectory Analysis	2	1	0	0
Other methods	4	2	2	1

Of the articles included (*n* = 107), the traditional method for RS detection Then Test registered the highest number of RS recalibration type (27), while Oort’s SEM registered a lesser number of studies detecting recalibration (24), but the highest record of reprioritization (13) and reconceptualization (7) of the entire systematic review (see Table 3). Schmidt’s SEM detected changes in internal standards only in two (2) articles. The rest of the methods also proved to be used for the detection of the three types of RS, except for the Relative Importance Methods, Mixed-Effects Regression, Random Forest Regression, and Latent Trajectory Analysis, which did not identify the presence of reconceptualization.

**Fig. 4:**
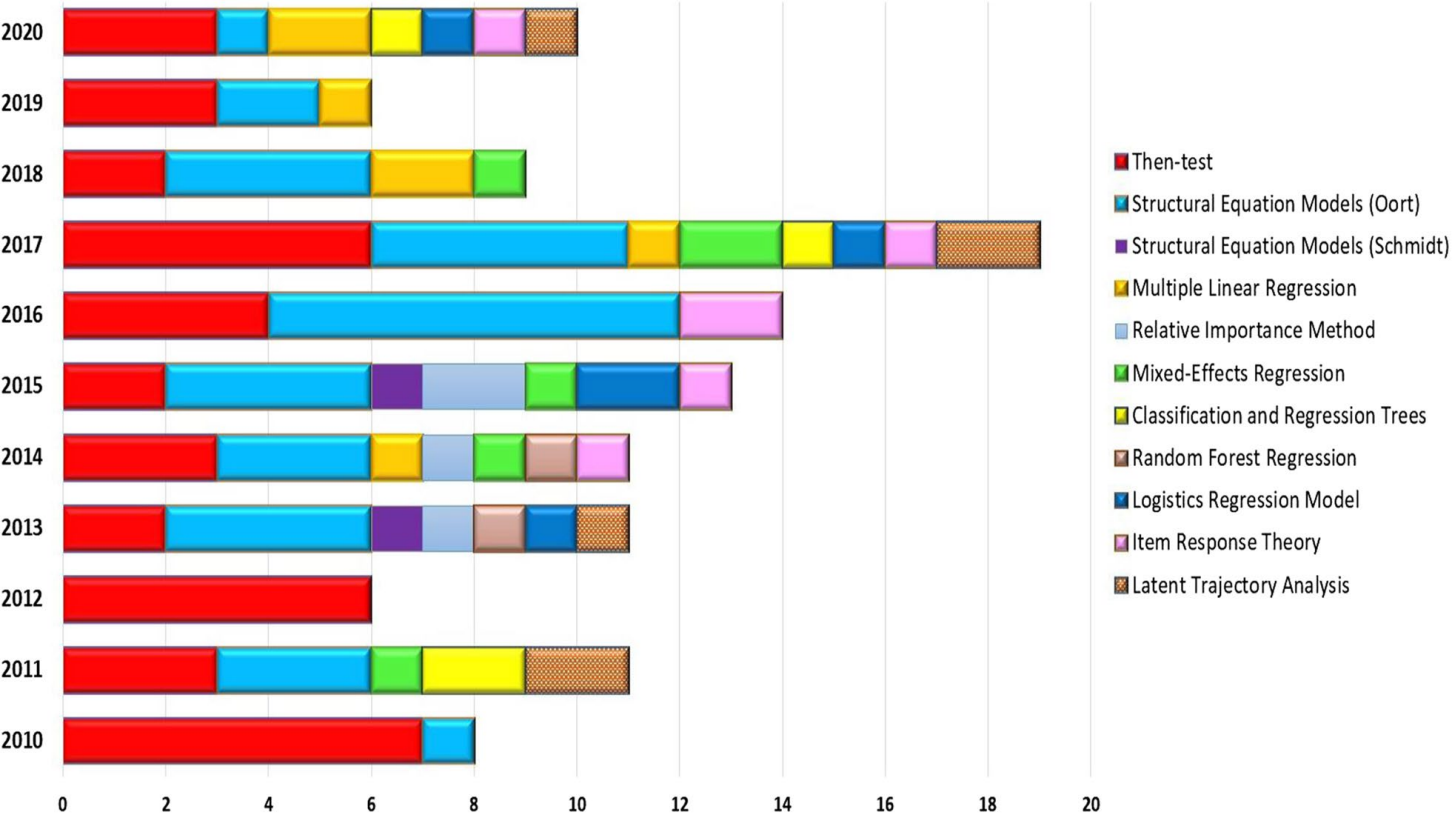
Frequency of methods used for Response Shift detection by year

Despite the effectiveness for detecting RS, this review evidences the growing interest for exploring different statistical methods for RS studies (see Fig. 4). The most frequently used methods were Then Test and Oort’s SEM between 2010 and 2020. However, during the last years, the figure shows that other alternative techniques are being considered for the assessment of this phenomenon.

## Discussion

After the systematic review, the study examined the advances on HRQOL research through different methods for the detection of RS. It describes how the detection of this phenomenon is being evaluated in recent years, which methods are used the most, and the most identified type of RS. For physicians and researchers, systematic reviews are useful sources of evidence and scientific advances [32], they provide elements so clinical policy-makers can evaluate risks, benefits and effects on health care, as well as new research initiatives [26, 27].

The majority of HRQOL studies and RS detection methods were published during 2016 and 2017. One third of the articles included in the review were studying cancer disease [33–41]. Then Test was the most used method mainly for primary data measuring recalibration [42–48]. Several studies remarked that Then Test may also identify other RS types: reprioritization y reconceptualization [33, 49, 50]. Similar results were presented in Sajobi et al. [15] study corresponding to a systematic review about RS detecting methods were 54.5% of the articles used Then Test for this purpose. As same, in Ilie et al. [23] a 60.0% of the cancer studies remarked using this methodology, but authors indicate that interpretation must be cautious as it is bias prone.

Then Test has the advantage for easy handling and analysis, but its disadvantage relies in random errors and/or confound and difficult for interpretation. Therefore, this appraisal suggests the utilization of individualized methods with strong statistical rigor [51].

Oort’s SEM [17] differs from Then Test, since it is used for studies based on primary and secondary data. This multivariate method evidences the capability for detecting changes in all type of RS: recalibration, reprioritization, and reconceptualization. Several studies reiterate Oort’s SEM as an effective method to detect changes in patients, despite the different type of RS or data used [52–54]. This detection capacity might be the reason for a larger interest for researchers of using this method.

Schmidt’s model was used in two studies detecting only recalibration. In comparative studies with Oort’s method [55, 56], only this type of RS is identified, but conclusions indicated that both approaches use different parameters to identify recalibration: Schmidt defines recalibration as the change in factor variances or factor loadings over time, while Oort by the change in intercepts.

Other methods obtained more records for RS detection: Mixed-Effects and Multiple Linear Regression [57–60], Classification and Regression Trees [22, 61], Item Response Theory [18, 62] and Relative Importance Methods [63–65].

Despite a major frequency of methods to detect recalibration, it calls the attention that all three studies using Relative Importance Method detected reprioritization [63–65]. Mixed-Effect Regression method [57, 58], Classification and Regression Trees [22, 61] and Latent Trajectory Analysis [66] neither provided information on reconceptualization.

During this study we observed that the sample size is related to the type of method used for RS detection. Then Test is more flexible and functional in small samples or in specific studies, while methods based on stronger statistic techniques require larger samples. According to Schwartz et al. [67], for using advanced multivariate methods capable to detect RS, data analysis must include sufficient individuals participating in the study, as well as considering certain number of parameters such a clear the model, loading factor, relationship between the items, data distribution, and processes for estimating these parameters.

The need of exploring new multivariate methodologies for analyzing this phenomenon brings an alternative proposal for studying HRQOL and RS detection through three-way data: dual STATIS Method [68, 69]. The proposal presented by Vicente Galindo [70] centers in a procedure integrating the dual STATIS methods and the comparison of Krzanowski [71] subspaces, and examinates factorial structures from multiple data sets focused to identify the existence of change.

The systematic reviews evaluated in this study [15, 23, 28–31] confirm the continuous use of the same methods for detecting RS and its different types, their advantages, and limitations. The previous proposal is an example of existing opportunities to continue examining other strong statistic methodologies allowing to deepen this line of investigation and reducing bias and analysis ambiguities.

## Conclusion

The systematic review has demonstrated to become an adequate and convenient methodology to identify and synthetize advances of specific topics. Results have evidenced a generalized interest for studying HRQOL and RS for different diagnostic groups. RS detection continue attracting researcher’s attention that have consolidated a set of methods for its analysis: Then Test, Oort’s SEM, Multiple Linear and Mixed-Effects Regressions, Item Response Theory and Latent Trajectory Analysis. Oort’s SEM becomes the most versatile method in its capability for detecting RS in all different types.

Results demonstrated that not all the methods achieve RS detection in similar proportions, mostly capable to identify recalibration, in some cases reprioritization, and in few studies reconceptualization. At the same time, previous systematic reviews and the result from this updating research conclude that same methods have been used during the last years and there is no evidence found of alternative statistical techniques proposed for detecting RS. Perhaps, our study states a need for exploring other methods with similar detection capacity, with robust and highly precise methodology either graphical oriented or with simpler methodologies.

Previous systematic reviews and the need to continue investigating about this phenomenon have motivated the update of this search within the last decade (2010–2020). Although these reviews have used a larger number of search terms including different RS types as well as data measurement characteristics, this systematic review has intentionally generalized the terms using RS and HRQOL to focus the results on those studies centered on this topic.

Since RS can be evaluated from different perspectives, and other disciplines such as statistics, psychology, humanities, and other related social sciences can provide significant contributions, it is recommended to continue studying the RS phenomenon, its detection, and types from a multidisciplinary approach.

## Supplementary Information

**Additional file 1:** List of articles included in the systematic review: 2010-2020.

## Data Availability

All data generated or analyzed during this study are included in this published article and its supplementary information files.

## Abbreviations

RS: Response Shift
HRQOL: Health-Related Quality of Life
SEM: Structural Equation Model
PRO: Patient-reported outcomes

## References

[1] World Health Organization [WHO]. WHOQOL: measuring quality of life. https://apps.who.int/iris/handle/10665/63482 (1997). Accessed 20 Aug 2020.

[2] Centers for Disease Control and Prevention [CDC]. Measuring healthy days: population assessment of health-related quality of life. https://www.cdc.gov/hrqol/pdfs/mhd.pdf (2000). Accessed 20 Aug 2020.

[3] Kang K, Gholizadeh L, Inglis SC, Han HR (2016). Interventions that improve health-related quality of life in patients with myocardial infarction. Qual Life Res.

[4] Shiroiwa T, Fukuda T, Shimozuma K (2017). Long-term health status as measured by EQ-5D among patients with metastatic breast cancer: comparison of first-line oral S-1 and taxane therapies in the randomized phase III SELECT BC trial. Qual Life Res.

[5] Howard G, Dailey P (1979). Response shift bias: a source of contamination of self-reporting measures. J Appl Psychol.

[6] Golembiewski RT, Billingsley K, Yeager S (1976). Measuring change and persistence in human affairs: types of changes generated by OD design. J Appl Behav Sci.

[7] Schwartz CE, Sprangers MA (1999). Methodological approaches for assessing response shift in longitudinal quality of life research. Soc Sci Med.

[8] Hamidou Z, Dabakuyo-Yonio T, Bonnetain F (2011). Impact of response shift on longitudinal quality of life assessment in cancer clinical trials. Expert Rev Pharmacoecon Outcomes Res.

[9] Sprangers MA, Schwartz CE (1999). Integrating response shift into health-related quality of life research: a theoretical model. Soc Sci Med.

[10] Rapkin BD, Schwartz CE (2004). Toward a theoretical model of quality-of-life appraisal: Implications of findings from studies of response shift. Health Qual Life Outcomes..

[11] McPhail S, Haines T (2010). Response shift, recall bias and their effect on measuring change in health-related quality of life amongst older hospital patients. Health Qual Life Outcomes.

[12] Crosby RD, Kolotkin RL, Williams GR (2003). Defining clinically meaningful change in health-related quality of life. J Clin Epidemiol.

[13] Kvam AK, Wisløff F, Fayers PM (2010). Minimal important differences and response shift in health-related quality of life; a longitudinal study in patients with multiple myeloma. Health Qual Life Outcomes.

[14] Bulteau S, Sauvaget A, Vanier A (2019). Depression reappraisal and treatment effect: will response shift help improve the estimation of treatment efficacy in trials for mood disorders?. Front Psychiatry.

[15] Sajobi TT, Brahmbatt R, Lix L, Zumbo B, Sawatzki R (2018). Scoping review of response shift methods: current reporting practices and recommendations. Qual Life Res.

[16] Schmidt N (1982). The use of analysis of covariance structures to assess beta and gamma change. Multivar Behav Res.

[17] Oort FJ (2005). Using structural equation modeling to detect response shifts and true change. Qual Life Res.

[18] Anota A, Bascoul-Mollevi C, Conroy T (2014). Item response theory and factor analysis as a mean to characterize occurrence of response shift in a longitudinal quality of life study in breast cancer patients. Health Life Outcomes.

[19] Guilleux A, Blanchin M, Vanier A (2015). RespOnse Shift ALgorithm in Item response theory (ROSALI) for response shift detection with missing data in longitudinal patient-reported outcome studies. Qual Life Res.

[20] Lix LM, Sajobi TT, Sawatzky R (2013). Relative importance measures for reprioritization response shift. Qual Life Res.

[21] Ahmed S, Mayo N, Scott S, Kuspinar A, Schwartz C (2011). Using latent trajectory analysis of residuals to detect response shift in general health among patients with multiple sclerosis article. Qual Life Res.

[22] Li Y, Schwartz C (2011). Data mining for response shift patterns in multiple sclerosis patients using recursive partitioning tree analysis. Qual Life Res.

[23] Ilie G, Bradfield J, Moodie L (2019). The role of response-shift in studies assessing quality of life outcomes among cancer patients: a systematic review. Front Oncol.

[24] Montori VM, Swiontkowski MF, Cook DJ (2003). Methodologic issues in systematic reviews and meta-analyses. Clin Orthop Relat Res.

[25] Moher D, Shamseer L, Clarke M (2015). Preferred reporting items for systematic review and meta-analysis protocols (PRISMA-P) 2015 statement. Syst Rev.

[26] Moher D, Liberati A, Tetzlaff J, Altman DG (2009). Preferred reporting items for systematic reviews and meta-analyses: the PRISMA statement. PLoS Med.

[27] Liberati A, Altman DG, Tetzlaff J (2009). The PRISMA statement for reporting systematic reviews and meta-analyses of studies that evaluate healthcare interventions: explanation and elaboration. BMJ.

[28] Nielsen LK, Jarden M, Andersen CL, Frederiksen H, Abildgaard NA (2017). Systematic review of health-related quality of life in longitudinal studies of myeloma patients. Eur J Haematol.

[29] Powden C, Hoch M, Hoch J (2018). Examination of response shift after rehabilitation for orthopedic conditions: a systematic review. J Sport Rehabil.

[30] Scholten AC, Haagsma JA, Steyerberg EW, vanBeeck EF, Polinder S (2017). Assessment of pre-injury health-related quality of life: a systematic review. Popul Health Metr.

[31] Hinds AM, Sajobi TT, Sebille V, Sawatzky R, Lix LM (2018). A systematic review of the quality of reporting of simulation studies about methods for the analysis of complex longitudinal patient-reported outcomes data. Qual Life Res.

[32] Manchikanti L, Datta S, Smith HS, Hirsch JA (2009). Evidence-based medicine, systematic reviews, and guidelines in interventional pain management: part 6. Systematic reviews and meta-analyses of observational studies. Pain Phys.

[33] Taminiau-Bloem E, van Zeuuren F, Koeneman M (2010). A ‘short walk’ is longer before radiotherapy than afterwards: a qualitative study questioning the baseline and follow-up design. Health Qual Life Outcomes.

[34] Neuman H, Park J, Fuzesi S, Temple L (2010). Rectal cancer patients’ quality of life with a temporary stoma: shifting perspectives. Dis Colon Rectum.

[35] Dabakuyo TS, Guillemin T, Conroy T (2013). Response shift effects on measuring post-operative quality of life among breast cancer patients: a multicenter cohort study. Qual Life Res.

[36] Brinksma A, Tissing WJ, Sulkers E, Kamps WA, Roodbol PF, Sanderman R (2014). Exploring the response shift phenomenon in childhood patients with cancer and its effect on health-related quality of life. Oncol Nurs Forum.

[37] Gerlich C, Schuler M, Jelitte M (2016). Prostate cancer patients’ quality of life assessments across the primary treatment trajectory: ‘True’ change or response shift?. Acta Oncol.

[38] Tessier P, Blanchin M, Sébile V (2017). Does the relationship between health-related quality of life and subjective well-being change over time? An exploratory study among breast cancer patients. Soc Sci Med.

[39] Aburub A, Gagnon B, Ahmed S, Rodríguez AM, Mayo N (2018). Impact of reconceptualization response shift on rating of quality of life over time among people with advanced cancer. Support Care Cancer.

[40] Preiß M, Friedrich M, Stolzenburg JU, Zenger M, Hinz A (2019). Response shift effects in the assessment of urologic cancer patients’ quality of life. Eur J Cancer Care.

[41] Murata T, Suzukamo Y, Shiroiwa T (2020). Response Shift-adjusted treatment effect on health-related quality of life in a randomized controlled trial of Taxane versus S-1 for metastatic breast cancer: structural equation modeling. Value Health.

[42] Dempster M, Carney R, McClements R (2010). Response shift in the assessment of quality of life among people attending cardiac rehabilitation. Br J Health Psychol.

[43] Shi HY, Lee KT, Lee HH (2011). Response shift effect on gastrointestinal quality of life index after laparoscopic cholecystectomy. Qual Life Res.

[44] Galenkamp H, Huisman M, Braam A, Deeg D (2012). Estimates of prospective change in self-rated health in older people were biased owing to potential recalibration response shift. J Clin Epidemiol.

[45] Rutgers M, Creemers L, Yang KG, Raijmakers N, Dhert W, Saris D (2015). Osteoarthritis treatment using autologous conditioned serum after placebo. Patient considerations and clinical response in a non-randomized case series. Acta Orthop.

[46] Arthur J, Watts T, Davies R, Manchaiah V, Slater J (2016). An exploratory study identifying a possible response shift phenomenon of the Glasgow hearing aid benefit profile. Audiol Res.

[47] Reissmann D, Erler A, Hirsch C, Sierwald I, Machuca C, Schierz O (2018). Bias in retrospective assessment of perceived dental treatment effects when using the Oral Health Impact Profile. Qual Life Res.

[48] Haagsma J, Spronk I, de Jongh M, Bonsel G, Polinder S (2020). Conventional and retrospective change in health-related quality of life of trauma patients: an explorative observational follow-up study. Health Qual Life Outcomes.

[49] Höfer S, Pfaffenberger N, Renn D, Platter M, Ring L (2011). Coronary intervention improves disease specific health-related quality of life but not individualised quality of life: a potential response shift effect?. Applied Res Qual Life.

[50] Machuca C, Vettore M, Krasuska M, Baker S, Robinson P (2017). Using classification and regression tree modelling to investigate response shift patterns in dentine hypersensitivity. BMC Med Res Methodol.

[51] Schwartz CE (2010). Applications of response shift theory and methods to participation measurement: a brief history of a young field. Arch Phys Med Rehabil.

[52] DeConde A, Bodner T, Mace J, Smith T (2014). Response shift in quality of life after endoscopic sinus surgery for chronic rhinosinusitis. JAMA Otolaryngol Head Neck Surg.

[53] Verdam MG, Oort FJ, Sprangers MAG (2017). Structural equation modeling-based effect-size indices were used to evaluate and interpret the impact of response shift effect. J Clin Epidemiol.

[54] Wang X, Xu X, Han H (2018). Using structural equation modeling to detect response shift in quality of life in patients with Alzheimer’s disease. Int Psychogeriatr.

[55] Gandhi PK, Ried LD, Huang IC, Kimberlin C, Kauf T, Suh DC (2011). Identification of Response Shift among hypertensive patients with coronary artery disease using two structural equation modeling techniques. Value Health.

[56] Gandhi PK, Ried LD, Huang IC, Kimberlin C, Kauf T (2013). Assessment of response shift using two structural equation modeling techniques. Qual Life Res.

[57] Lacey H, Loewenstein G, Ubel P (2011). Compared to what? A joint evaluation method for assessing quality of life. Qual Life Res.

[58] Schwartz C, Quaranto B, Rankin B, Healy B, Vollmer T, Sprangers M (2014). Fluctuations in appraisal over time in the context of stable versus non-stable health. Qual Life Res.

[59] Nichols G, Antoun J, Fowler P, Al-Ani A, Farella M (2018). Long-term changes in oral health-related quality of life of standard, cleft, and surgery patients after orthodontic treatment: a longitudinal study. Am J Orthod Dentofac Orthop.

[60] Felix J, Becker C, Vogl M, Buschner P, Plötz W, Leidi R (2019). Patient characteristics and valuation changes impact quality of life and satisfaction in total knee arthroplasty – results from a German prospective cohort study. Health Qual Life Outcomes.

[61] Machuca C, Vettore M, Robinson P (2020). How peoples’ ratings of dental implant treatment change over time?. Qual Life Res.

[62] Blanchin M, Sébille V, Guilleux A, Hardouin J (2016). The Guttman errors as a tool for response shift detection at subgroup and item levels. Qual Life Res.

[63] Schwartz C, Sajobi T, Lix L, Quaranto B, Finkelstein J (2013). Changing values, changing outcomes: the influence of reprioritization response shift on outcome assessment after spine surgery. Qual Life Res.

[64] Sajobi TT, Fiest KM, Wiebe S (2014). Changes in quality of life after epilepsy surgery: The role of reprioritization response shift. Epilepsia.

[65] Sajobi TT, Lix L, Singh G, Lowerison M, Engbers J, Mayo N (2015). Identifying reprioritization response shift in a stroke caregiver population: a comparison of missing data methods. Qual Life Res.

[66] Salmon M, Blanchin M, Rotonda C, Guillemin F, Sebille V (2017). Identifying patterns of adaptation in breast cancer patients with cancer-related fatigue using response shift analyses at subgroup level. Cancer Med.

[67] Schwartz C, Ahmed S, Sawatzky R (2013). Guidelines for secondary analysis in search of response shift. Qual Life Res.

[68] L’Hermier des Plantes H. Structuration des tableaux á trois indices de la statistique. Théorie et applications d’une méthode d’analyse conjointe. 1976. Thése 3° cycle, USTL, Montpellier.

[69] Escoufier Y (1976). Opérateur associé á un tableau de données. Annales de L’Insee.

[70] Vicente-Galindo P. Contribuciones al análisis de datos de calidad de vida relacionada con la salud. 2003. Doctoral tesis, University of Salamaca, Spain.

[71] Krzanowski WJ (1979). Between-groups comparison of principal components. J Am Stat Assoc.

