# Supplementary material for: Detection of response shift in health-related quality of life studies: a systematic review
Source: Health Qual Life Outcomes. 2022 Feb 5;20:20. doi: 10.1186/s12955-022-01926-w (PMC8818219; doi:10.1186/s12955-022-01926-w)
Supplement: Supplementary file 1 — Additional file 1: List of articles included in the systematic review: 2010-2020. [file 12955_2022_1926_MOESM1_ESM.pdf]

Author	Country	Year	Speciality of the study	Data type	Sample size (initial)	Sample size (end)	Population Type (Specific/Mixed)	Questionnaire	Yes/No (Response Shift)
Dempster, M. *et al.*	Northern Irland	2010	Cardiology	Primary	84	57	Mixed	SEIQOL-DW	Yes
Ito, N. *et al.*	Japan	2010	Gastroenteretomy	Primary	20	13	Mixed	SF-36	Yes
Kievit, W. *et al.*	Netherland	2010	Arthritis	Primary	212	197	Mixed	DAS28 / VAS	Yes
Kvam, A. *et al.*	Norway	2010	Oncology	Primary	260	239	Mixed	EORTC QLQ-C30	Yes
McPhail, S. & Terry, H.	Australia	2010	General Medicine	Primary	103	101	Mixed	EQ-5D / VAS	Yes
Razmjou, H. *et al.*	Canada	2010	Orthopedics	Primary	107	107	Mixed	ASES-36	Yes
Taminiau-Bloem, E. *et al.*	Netherland	2010	Oncology	Primary	92	50	Mixed	EORTC QLQ-C30	Yes
King-Kallimanis, B. *et al.*	Netherland	2010	HIV Patients	Primary	403	403	Mixed	HAART	Yes
Ahmed, S. *et al.*	Canada	2011	Neurology	Secondary	1566	1312	Mixed	SF-12	No
Hinz, A. *et al.*	Germany	2011	Urology	Primary	427	275	Male	PHQ-2.	No
King-Kallimanis, B. *et al.*	Netherland	2011	Neurology	Secondary	2952	1767	Mixed	NARCOMS/SF-12	No
Li, Y. & Schwartz, CE.	United States	2011	Neurology	Secondary	3839	3008	Mixed	NARCOMS/SF-12	Yes
Schwartz, CE. *et al.*	United States	2011	Neurology	Secondary	3008	3008	Mixed	NARCOMS/ SF-12	Yes
Hon-Yi, Shi. *et al.*	Taiwan	2011	Gastroinstestinal	Primary	359	353	Mixed	GIQLI	Yes
Höfer, S. *et al.*	Austria	2011	Cardiology	Primary	64	64	Mixed	SEIQoL-DW y MacNew	Yes
Lacey, H.P. *et al.*	United States	2011	Obesity	Primary	1865	1865	Mixed	Encuesta por Internet	Yes
Barclay-Goddar, R. *et al.*	Canada	2011	Neurology	Secondary	677	677	Mixed	SF-36	Yes
Galenkamp, H. *et al.*	Netherland	2012	Gerontology	Primary	1992	1274	Mixed	SRH-T1 y SRH-T2	Yes
Kimura, A. *et al.*	Japan	2012	Oral Health	Primary	173	138	Mixed	OHRQoL	Yes
Neuman, H. *et al.*	United States	2012	Oncology	Primary	60	60	Mixed	EORTC -30; EORTC-38 y SQOLS8	Yes
Reissmann, D. *et al.*	Germany	2012	Ortodoncy	Primary	126	126	Mixed	OHIP-G y OHIP-G49	Yes
Zhang, X. *et al.*	Singapur	2012	Orthopedics	Primary	74	74	Mixed	SF-36 / EuroQoL	Yes
Schwartz, CE. *et al.*	United States	2012	Inmunology	Primary	521	521	Mixed	QOL Appraisal Profile/Rand-36 /General Health Then Test	No
Visser, M. *et al.*	Netherland	2013	Oncology	Primary	202	202	Mixed	SF-36	Yes
Arons, A. *et al.*	Netherland	2013	Psychiatry	Primary	220	151	Mixed	VAS	Yes
Boucekine, M. *et al.*	France	2013	Neurology	Secondary	580	524	Mixed	MusiQoL y SF-36	Yes
Dabakuyo, T.S. *et al.*	France	2013	Oncology	Primary	381	381	Female	QLQ-C30, BR23 y EurQOL-EQ-5D	Yes
Fokkema, M. *et al.*	Netherland	2013	Psychiatry	Primary	560	250	Mixed	BDI	Yes

Gandhi, P. et al.	United States	2013	Cardiology	Secondary	2317	909	Mixed	SF-36	Yes
Gandhi, P. et al.	United States	2013	Cardiology	Secondary	2317	788	Mixed	SF-36	Yes
Lix, L. et al.	Canada	2013	Gastroinstestinal	Primary	388	388	Mixed	EII (IBDQ) y SF-36	No
Schwartz, CE. & Sajobi, T.T.	United States	2013	Orthopedics	Primary	178	169	Mixed	SF-36 /ODI	Yes
Edelaar-Peeters, Y. & Stiggelbout, A.	Netherland	2013	Neurology	Primary	44	44	Mixed	VAS	Yes
Flynn, T. et al.	Australia	2013	Gerontology	Primary	478	315	Mixed	ICECAP-O	No
Ahmed, S. et al.	Canada	2014	Arthritis, heart failure, diabetes, lung deficiency	Secondary	776	776	Mixed	SF-36	No
Anotta, A. et al	France	2014	Oncology	Secondary	381	381	Female	EORT QLQ-C30/EORT QLQ-BR23	Yes
Barclay-Goddar, R. & Tate, R.	Canada	2014	Cardiology	Secondary	3983	745	Male	SF-36	Yes
Brinksman, A. et al	Netherland	2014	Oncology	Primary	61	37	Mixed	PedsQL / Cantril ladder	Yes
DeConde, A. et al.	United States	2014	Otolaryngology	Primary	514	514	Mixed	SNOT-22	Yes
Hamidou, Z. et al.	France	2014	Oncology	Primary	381	381	Female	EORTC-QLQ-30 Y BR-23	Yes
Howard, J. et al.	United States	2014	Orthopedics	Primary	56	48	Mixed	SF-36	No
Sajobi, T.T. el at.	Canada	2014	Neurology	Primary	80	80	Mixed	QOLIE-31	Yes
Schwartz, C.E. et al.	United States	2014	Neurology	Secondary	859	859	Mixed	NARCOMS/SF-12	Yes
Elliot, B. et al.	United States	2014	Nefrology	Primary	31	27	Mixed	Qualitative Study	Yes
Blanckenburg, P. et al.	Germany	2014	Oncology	Primary	480	86	Mixed	WHOQOL-BREF	Yes
Boucekine, M. et al.	France	2014	Psychiatry	Primary	124	124	Mixed	SF-36	Yes
Guilleux, A. et al.	France	2015	Different diagnosis	Secondary	669	537	Mixed	SF-36	Yes
Liu, J. & Davis, G.	United States	2015	Otolaryngology	Primary	79	32	Mixed	SNOT-20	Yes
Mayo, N. et al.	Canada	2015	Internal Medicine	Secondary	388	388	Mixed	SF-36	No
Mollerup A. & Johansen, J.	Denmark	2015	Dermatology	Primary	306	306	Mixed	VASnow / VASworst	No
Rutgers, M. et al.	Netherland	2015	Orthopedics	Primary	74	20	Mixed	VAS	Yes
Sajobi, T.T. et al.	Canada	2015	Neurology	Secondary	409	409	Mixed	SF-36	Yes
Traa, M. et al.	Netherland	2015	Oncology	Primary	672	205	Mixed	WHOQOL-Bref	No
Verdam, M. et al.	Netherland	2015	Oncology	Primary	1157	1157	Mixed	EQ-5D/ EORTC-QLQ-C30 / RSCL	Yes
Phillips, R. et al.	Singapur	2015	Oncology	Primary	167	167	Mixed	EORTC QLQ-C30	No
Schwartz, C.E. et al.	Canada	2015	chronic illness	Primary	172	172	Mixed	SF-36y VAS	No

Blanchin, M. *et al.*	France	2016	chronic illness	Primary	669	499	Mixed	SF-36	Yes
Gandhi, P. *et al.*	United States	2016	Otolaryngology	Primary	238	238	Mixed	PAQLQ	No
Gerlich, G. *et al.*	Germany	2016	Oncology	Primary	522	402	Male	EORTC QLQ-C30	Yes
Hollman, F. *et al.*	Netherland	2016	Orthopedics	Primary	36	36	Mixed	WORC /EuroQol -5D-3L	No
Lix, L. *et al.*	Canada	2016	Internal Medicine	Primary	606	388	Mixed	SF-36	Yes
Nolte, S. *et al.*	Germany	2016	Psychiatry	Secondary	1188	1188	Mixed	ICD-10	Yes
Ousmen, A. *et al.*	France	2016	Oncology	Secondary	381	381	Female	EORTC-QLQ-C30/EORTC-QLQ-BR23	Yes
Reissmann, D. et al.	Germany	2016	Oral Health	Secondary	554	554	Mixed	OHIP	No
Sebille, V. *et al.*	France	2016	Internal Medicine	Primary	390	390	Mixed	PreKit-QOL	No
Taminiau-Bloem, E. *et al.*	Netherland	2016	Oncology	Primary	50	24	Mixed	EORTC QLQ-C30	No
Verdam, M. *et al.*	Netherland	2016	Oncology	Secondary	485	437	Mixed	SF-36	Yes
Wu, P.	Taiwan	2016	Psychology	Primary	320	320	Mixed	BDII	Yes
Arthur, J. *et al.*	United Kingdom	2016	Audiology	Primary	16	16	Mixed	GHABP	Yes
Jakola, A. *et al.*	Sweden	2017	Neurology	Primary	210	73	Mixed	EQ-5D-3L	No
Chen, P. et al.	United States	2017	Mental health	Primary	114	114	Mixed	ISI and PSQI	No
Hosseini, K. *et al.*	France	2017	Internal Medicine	Primary	264	171	Mixed	EQ-5D-3L y SF-6D	No
Jabrayilov, R. *et al.*	Netherland	2017	Psychiatry	Secondary	540	450	Mixed	OQ-45	No
Machuca, C. *et al.*	Germany	2017	Oral Health	Primary	75	75	Mixed	DHEQ /CRT	Yes
Powell, G. *et al.*	Canada	2017	Mental health	Secondary	2148	2148	Mixed	QOLI-20	No
Sajobi, T.T. *et al.*	Canada	2017	Pediatrics	Primary	373	373	Mixed	QOLCE-55	Yes
Salmon, M. *et al.*	France	2017	Oncology	Secondary	466	466	Female	MFI-20 / QLC-C30	Yes
Schwartz, C. E. *et al.*	United States	2017	Neurology	Secondary	858	858	Mixed	Rand-12	Yes
Hinz, A. *et al.*	Alemania	2017	Oncology	Secundary	2059	2059	Mixed	EORTC QLC-C30	Do not indicate
Spulling, S. *et al.*	Germany	2017	Gerontology	Primary	3854	1764	Mixed	SRH-T1 y SRH-T2	Yes
Tessier, P. *et al.*	France	2017	Oncology	Secondary	215	215	Female	SWLS y EORTC QLQ-C30	Yes
Verdam, M. *et al.*	Netherland	2017	Oncology	Primary	170	170	Mixed	SF-36	Yes
Yang, Y. *et al.*	United States	2017	Neurology	Primary	124	124	Mixed	EQ-5D/UPDRS	No
Hosseini, B. et al.	Iran	2017	Oncology	Primary	211	211	Mixed	EORTC QLQ-C30	Yes

Aburub, A. *et al.*	Canada	2018	Oncology	Primary	192	97	Mixed	EQ-5D	Yes
Friedrich, M. *et al.*	Germany	2018	Oncology	Primary	350	308	Female	EORTC QLQ-C30	Yes
Krägeloh, C. *et al.*	New Zealand	2018	Psychology	Primary	181	181	Mixed	CHIME	Yes
Nichols, G. *et al.*	New Zealand	2018	Ortodoncy	Primary	57	57	Mixed	OHIP-14	Yes
Reissmann, D. *et al.*	Germany	2018	Oral Health	Primary	126	126	Mixed	OHIP -49	Yes
Murray, A. *et al.*	United Kingdom	2018	Psychology	No especificado	359	359	Mixed	CORE-OM	Yes
Schmidt, H., *et al.*	Germany	2018	Oncology	Secondary	518	518	Mixed	EORTC QLC-30	No
Schwartz, C.E. *et al.*	United States	2018	Neurology	Secondary	2104	2104	Mixed	SF-36	Yes
Wang, X. *et al.*	China	2018	Gerontology	Primary	238	238	Mixed	SF-12	Yes
Carlier, I. *et al.*	Netherland	2019	Psychiatry	Primary	206	206	Mixed	SQ-48	Yes
Felix, J. *et al.*	Germany	2019	Orthopedics	Primary	203	137	Mixed	EQ-5D-3L	Yes
Friedrich, M. *et al.*	Germany	2019	Cardiology	Primary	479	282	Mixed	EORTC-QLQ-C30	Yes
Powden, C. *et al.*	United States	2019	Orthopedics	Primary	20	20	Mixed	FAAM-Sport	No
PreilB, M. *et al.*	Germany	2019	Oncology	Primary	197	197	Male	EORTC QLQ-C30 y PHQ-4	Yes
Rohn, E. *et al*	United States	2019	Neurology	Primary	40	40	Mixed	SCI-QOL	Si
Haagsma, J. *et al.*	Netherland	2020	Traumalogy	Primary	550	550	Mixed	EQ-5D-3L y EQ-VAS	Yes
Machuca, C. *et al.*	United Kingdom	2020	Ortodoncy	Primary	145	145	Mixed	OHIP-Edent	Yes
Schwartz, C.E. *et al.*	United States	2020	General Medicine	Secondary	1481	1481	Mixed	PROMIS-10	No
ten Ham, R. *et al.*	United States	2020	Oncology	Primary	3161	1677	Male	SF-36 y UCLA (PCI)	Yes
Tew, M. *et al.*	Australia	2020	Orthopedics	Secondary	1892	1553	Mixed	SF-12	No
Topp, J. *et al.*	United States	2020	Neurology	Primary	100	93	Mixed	SF-12	No
Artavia-Mora, L. *et al.*	Netherland	2020	Virology	Secondary	3625	3625	Mixed	CD4	No
Murata, T. *et al.*	Japan	2020	Oncology	Secondary	368	368	Female	EORTC QLQ-C30	Yes
